# The ghost sex-life of the paedogenetic beetle *Micromalthus debilis*

**DOI:** 10.1038/srep27364

**Published:** 2016-06-07

**Authors:** M. Alejandra Perotti, Daniel K. Young, Henk R. Braig

**Affiliations:** 1 School of Biological Sciences, University of Reading, Reading, RG6 6AS, UK; 2 Department of Entomology, 1630 Linden Drive, University of Wisconsin, Madison, WI 53706, USA; 3 School of Biological Sciences, Bangor University, Bangor, LL57 2UW, UK

## Abstract

Genetic and sexual systems can be evolutionarily dynamic within and among clades. However, identifying the processes responsible for switches between, for instance, sexual and asexual reproduction, or cyclic and non-cyclic life histories remains challenging. When animals evolve parthenogenetic reproduction, information about the sexual mating system becomes lost. Here we report an extraordinary case where we have been able to resurrect sexual adults in a species of beetle that reproduces by parthenogenetic paedogenesis, without the production of adults. Via heat treatment, we were able to artificially induce adult beetles of *Micromalthus debilis* in order to describe its pre-paedogenetic mating system. Adults showed a highly female biased sex ratio, out-breeding behaviour, and sex-role reversal. Paedogenetic larvae of *Micromalthus* are infected with the endosymbiotic bacteria *Rickettsia* and *Wolbachia*. Clear signs of vestigialization in adults are concurrent with the loss of adults. Our data suggest an ancient female sex ratio bias that predates the loss of adults, perhaps associated with endosymbionts. We propose a model for the transition from a haplodiploid cyclical parthenogenetic life history to parthenogenetic paedogenesis. Paedogenetic development induces a new mechanism of sex ratio bias in midges, wasps and beetles.

Insects display an extraordinary richness of genetic and sexual systems. At least eight genetic forms of thelytokous parthenogenesis have been described, haplodiploidy is dominant in solitary and social Hymenoptera, hermaphroditism in scale insects, androgenesis is widespread in stick insects, and no less than four variations of hybridogenesis have been reported in ants^1^,^2^. As such, we now have a growing appreciation that genetic and sexual systems are far more dynamic than once thought. Attempts to provide a theoretical framework for explaining the evolution of genetic and sexual systems have revolved around the ideas of genomic conflicts, including those arising from selfish genetic elements, particularly between hosts and their endosymbionts^3^,^4^,^5^,^6^.

However, testing such hypotheses remains a major challenge. First, many of the species of interest share suites of characters that are often highly correlated (such as inbreeding, skewed sex ratios, or the presence of endosymbionts), making it difficult to identify cause and effect or eliminate third-variable problems such as the combination of two or more selfish genetic elements in one host^7^. Second, many of these traits make the organisms cryptic or hard to study by conventional means; they are typically not conventional “lab-rat” insects. Third, some species are monotypic, they are the only species in a genus or even family, making formal comparative analyses difficult or hard to interpret. Finally, all three of these challenges together make reconstructing the order in which traits evolved–crucial to disentangling cause and effect–extremely difficult.

Understanding the shifts to parthenogenesis can be particularly problematic, as asexuality by definition removes many of the traits associated with the mating system–including males–all at once. This can make reconstructing ancestral states problematic, compounding the difficulties of assigning cause and effect.

Here we report a remarkable experiment in which we recreated the lost adult stages in the beetle *Micromalthus debilis* LeConte, an insect with a bizarre sexual system.

*Micromalthus* is considered native to North America^8^, and is the only extant species of the ancient family Micromalthidae. The larvae bore in wood and are associated with fungal near-red-rot. It combines haplodiploidy, uni-oviparity for males (one male egg is laid at a time), poly-viviparity for females (several female larvae are delivered at a time), and internal and external matriphagy (consumption of the mother by the offspring). Larvae display sexual dimorphism. *Micromalthus* reproduces exclusively by larval thelytoky or paedogenesis (parthenogenetic reproduction of the female larval stage) (Fig. 1), and males are generally absent^9^,^10^,^11^,^12^,^13^. Despite its extraordinary features, there is a huge gap in research on *Micromalthus*. Only five experiments have been carried out in the past 100 years^9^,^10^,^11^,^12^,^13^ with the most recent on surveys of its associated microflora^14^. Constraints associated with working on this species range from its microscopical size and the uncertainty in finding wild colonies, to the dependency on micromanipulation of wood fragments to isolate living specimens.

Some authors have based their understanding of the *Micromalthus* life cycle on an opinion paper, assuming the adults are fertile^15^. The entire experimental literature, however, states that adults are sterile. Pollock and Normark, for example, reported that adults mate based on an incorrect observation of Barber^12^. Indeed, Barber observed two adults mounting, but after careful examination of the specimens by an expert in aedeagi (male intromittent organ) of beetles, both individuals were confirmed to be female. Barber was therefore the first who observed female-female mounting in beetles^12^,^16^.

This is the first experiment aiming to quantify *Micromalthus* ghost adults and their sex ratios and unravel clues on their past mating practices. To begin piecing together the ancestral mating system of *Micromalthus*, we used heat-treatment that led to the production of adults. We were interested in addressing several questions. What are the sex ratios in paedogenetic larvae and in heat-treated adults of *Micromalthus*? Sex ratio can be critical in hypothesizing the ancestral mating system. Strongly female-biased adult-operational-sex ratios are indicative of inbreeding and local mate competition^17^. Is there any observable mating behaviour expressed by the “resurrected” adults? Are heat-induced adults fit to mate? Mating behaviour can inform us about the level of mating competition, sex role observations and the extent of mating with kin. Finally, to what degree is *Micromalthus* associated with intracellular sex ratio distorters? Endosymbionts have been shown to play a role in shifts between genetic and sexual systems and in shaping host behaviour.

## Results

### Sex ratios of paedogenetic larvae

One fully examined colony contained 5,140 mature cerambycoid larvae and occupied a volume of ~25 cm^3^. The sex ratio of paedogenetic larvae of the 2 cm^3^ subsamples was highly female biased. Male producer larvae were rarely found and only a single egg was recovered. In all instances, the sex ratios were massively biased toward females (Binomial test, P < 0.001; Table 1 and S1).

Heat treatment did not influence larval sex ratios. For all 36 subsamples of paedogenetic populations not exposed to HT (including controls), the mean sex ratio was 0.0043 (SD = 0.005) (generalized linear model: Wald *χ*^*2*^_*1*_ = 794.747, P < 0.001). Sex ratios Before and After HT (Sets 1 and 2) were not significantly different, neither by location (Table S1)(Wilcoxon Signed Rank test, P = 0.678; and for normal distribution, Kolmogorov-Smirnov test, *Z*_*11*_ = 0.941, P = 0.283; and *t*_*11*_ = 0,313, P = 0.760). However, there was a significant sex ratio difference in Before and After HT between Locations 2 and 3 (generalized linear model: Wald *χ*^*2*^_*1*_ = 159, P < 0.001).

Heat treatment did have a very detrimental impact on the number of larvae, killing the great majority of cerambycoids.

### Sex ratios of ghost adults

Heat and drought treatment resulted in a slight decrease of the extremely female bias: 1,000 females, 59 males (Table 2). Nine individually counted populations yielded a maximum of 200 and a minimum of nine adults/population (patch). The mean sex ratio across 75 adult-emergence days averaged 0.036 (SD = 0.14; N = 75), clearly all highly significantly different to 0.5 (all P < 0.001). Analyses from all four locations also showed a highly female bias, significantly different to 0.5 (Table 2), albeit with significant heterogeneity across the four locations (*χ*^*2*^_*3*_ = 61.17, *LR*_*3*_ = 64.52; P < 0.001). Comparisons of larval and adult sex ratios used the only datasets available for paired comparisons across locations, and no difference was found in sex ratio (P = 0.109; Table S2).

During the behavioural experiments it was found that females rejected ‘related’ males - males from the same patch; therefore, the operational sex ratio was corrected to an “outbreeding operational sex ratio” (OOSR). The resulting OOSR was 0.057 (SD = 0.014), still highly female biased. Pre-mating dispersal involved males flying a short distance towards another nearby population with ‘unrelated’ (other patch) females (data not shown).

Each day, males and females emerged synchronously. The emergence period never lasted more than 45 minutes, with adult production commencing only when a peak temperature of 55 °C was reached. From a single colony, all emerging females walked to the most exposed area. They moved as a group, constantly walking and touching each other with their antennae (Movie S1). Most males displayed their aedeagus as soon as they emerged from the wood. They were unfit, sick or incapacitated; only 17 of 59 adult males matched the fitness of the majority of females and these 17 were selected for the behaviour experiments (Table S3).

### Mating behaviour of ghost adults

Adult male and female *Micromalthus* exhibited a range of sexual behaviours. Females preferred to mate with males from logs other than their natal log. Most notably, in the mixed sex groups (Same Patch and Unrelated), the patterns of sexual behaviour suggested sex-role reversal. Females initiated mating with unrelated males, competed for access, and actively mounted males. Females mounted the male, sometimes piling with up to five females at a time. In such cases, females tried to dislodge the others with their mandibles, which are otherwise used to bore tunnels in the wood. Damage to a male’s genitalia was observed once. Two females, larger than the male, everted a grasping organ formed from a pair of sharp valves located at the edge of the muscular *bursa copulatrix* (Fig. S1). The valves could move to grasp the aedeagus. Damage to the male occurred after a failed copulation attempt when the female dismounted the male while still holding on to part of his genitalia; consequently, the male lost one of its parameres.

Sexual behaviour significantly varied amongst the three experimental settings: females from the same patch, females from unrelated patches, and control females (Fig. 2). When males were present (Experiment 1: Same Patch; Experiment 2: Unrelated), females were far more likely to reject males (behaviour A) coming from the Same Patch (likelihood ratio test: *LR*_*1*_ = 275.91, *P* < 0.001); females were also more likely to mount (behaviour D) an Unrelated male (*LR*_*1*_ = 206.68, *P* < 0.001). However, female dance (behaviour C) was more likely to occur with males from the Same Patch (*LR*_*1*_ = 47.71, *P* < 0.001). Across all three experimental settings, the occurrence of female-female mounting (behaviour B) did not vary with the presence or absence of males (*LR*_*2*_ = 1.74, *P* = 0.42) whilst the occurrence of female-female fights (behaviour E) did increase if males were present and those males were from a different patch (*i.e.* the most favoured males; *LR*_*2*_ = 176.96, *P* < 0.001; Fig. 2).

Female-male mounting (behaviour D) differed between localities 2 and 3 (GLMM, *Z*_*1*_ = 4.46, *P* < 0.001), while female-female fights (behaviour E) showed significantly different performances between three localities (GLMM, L2 *Z*_*1*_ = −3.25, *P* < 0.001; L3 *Z*_*1*_ = 3.94, *P* < 0.001; L5, *Z*_*1*_ = 3.25, *P* < 0.001).

Adults were unable to copulate, and none of the 1,000 adult females was able to lay an egg or to produce progeny, either by sexual or parthenogenetic reproduction.

### Life span, anatomy and physiology of ghost adults

The average life span of adult females was 148.22 hours; approximately six days (SD = 23.43, N = 23) and much shorter in males 12.92 hours (SD = 10.58, N = 12).

All adult females (N = 1,000) and males (N = 59) were investigated for any signs of developmental abnormalities or vestigialization. Externally, deformed antennomeres were observed in five individuals and widespread asymmetry (supernumerary antennomeres) was observed in 20 of 60 females. Internally, the majority of organs of 265 specimens (¼ of adults) were reduced to not more than one third of their original size when fully developed or were completely missing, such that the thorax and abdomen looked nearly empty and transparent under a stereomicroscope. Nine of the empty females looked disabled, showing no movement and all died within 24 hours. When dissected, the thorax and abdomen of empty individuals (N = 10) were virtually devoid of organs. Many carried only a thin layer of what resembled fat body and the paired neural cord, both attached to the integument.

In all dissected females that exhibited no apparent degeneration, the spermatheca (receptacle where sperm is stored) was either reduced, vestigial, or absent altogether. Oocytes did not reach maturation in any of the females, including those that had mounted males.

### Endosymbiotic bacteria

Two major endosymbionts were found and they varied in prevalence. Our rDNA 16S and *wsp*A analyses identified *Wolbachia pipientis* in three of 62 larvae, while rDNA 16S analysis identified *Rickettsia bellii* in all larvae examined. This was confirmed by FISH analysis in cerambycoid larvae of *Micromalthus* (Fig. S2). No endosymbiont was amplified by PCR or observed by FISH in the ovaries of the dissected adult females, all of which were induced by the heat treatment.

## Discussion

**Figure 1:**
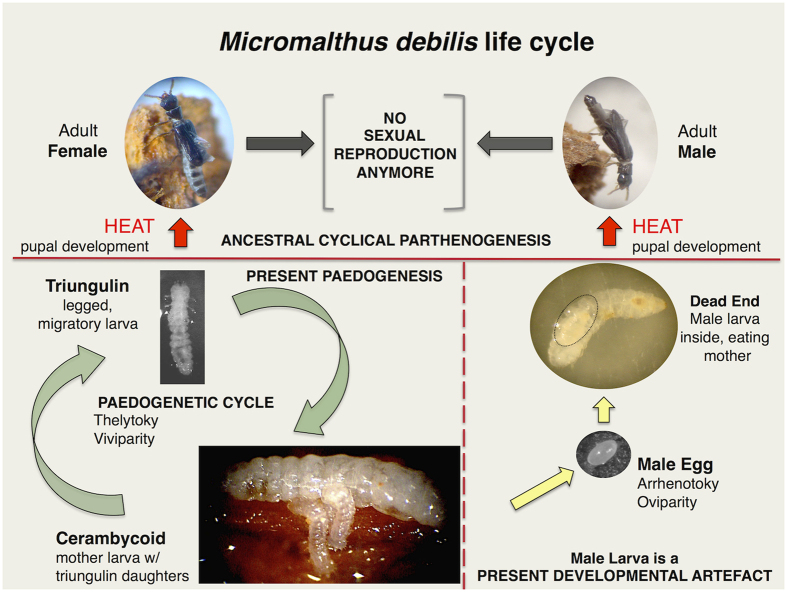
Evolution of the life cycle of *M. debilis*. Bottom left: green arrows show the present time obligate paedogenetic life cycle, where viviparous 1^st^ instar female larvae or triungulins are the legged, migratory stage. Bottom right: yellow arrows indicate rare, relic development of a male larva, where the oviparous 1^st^ instar male larva is legless. The male larva is under present conditions a developmental dead end. Top: red arrows show the rare development of adults induced through exposure to extreme heat. Adults are no longer reproductively functional; they were part of the ancestral cycle of sexual and asexual reproduction.

**Figure 2:**
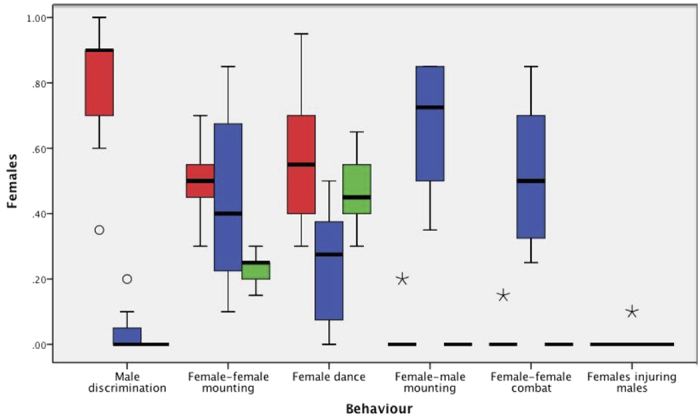
Normalized boxplot showing the portion of females of the three groups, Same Patch (red), Unrelated (blue), and Control (green) performing each behaviour. No female in the group Same Patch displayed Female dance, Female-male mounting, Female-female combat or Female injuring males. Outliers are identified as small circles (1.5 × interquartile range), whereas extreme values (3 × interquartile range) are marked with a star.

We were able to induce the production of adults in the paedogenetic beetle *Micromalthus debilis*. Heat treatment can lead to the production of adults, which are otherwise rarely seen in natural and laboratory populations. Heat treatment was originally suggested by Barber^12^; later Scott^11^,^18^ experimented on colonies and obtained only a few larval males and adult females. We were able to induce both female and male adults. Heat treatment is a universally recognized method to override mutations in viruses, prokaryotes and eukaryotes^19^,^20^,^21^. Phenotype and sometimes life cycle attributes change between a normal low, permissive temperature and a defined high, non-permissive temperature. The molecular mechanism that regulates the formation of adults in *Micromalthus* might have acquired missense mutation(s) that now prevent the formation of adults under physiological or permissive temperatures. *Micromalthus* is the first paedogenetic insect species that has been systematically exposed to defined high temperature regimes. We think it plausible that the metamorphosis from larvae to adults is controlled by a very limited number of genes that mutated. The precocious activation of the ecdysone receptor and ultraspiracle in paedogenetic gall midge larvae are sufficient to induce the development of ovaries within the larval instars^22^. Metamorphosis being regulated by very few genes would facilitate the selection for the paedogenetic phenotype. One could argue that high temperature, for example at the height of summer, might have in the past been a natural trigger for *Micromalthus* to change from the parthenogenetic part of its cycle to the sexual part. Although this cannot be ruled out, increased temperature is known to inhibit metamorphosis of butterfly and beetle larvae rather than to induce or accelerate it^23^,^24^. The temperature now required to induce adult development is so high that it causes severe mortality in the larvae. On average, only one in 315 cerambycoid larvae survived the heat treatment. At least 650 cerambycoid larvae were required to obtain a single adult female, while 10,000 cerambycoid larvae were estimated to be necessary for a single adult male to develop. The temperature required for adult development is clearly non-permissive and adults are no longer a physiological part of the life cycle (Fig. 1). Our studies support *Micromalthus* as a paedogenetic species in agreement with earlier experimental work^9^,^10^,^11^,^12^,^13^. These findings are in conflict with Caillol, Pollock and Normack, and Normack who proposed in opinion papers that it is a cyclically parthenogenetic species^15^,^25^,^26^.

### How informative are *Micromalthus* sex ratio biases?

In untreated paedogenetic larvae, females vastly predominate and this is the case for all locations. The sex ratio of larvae before and after heat treatment did not change significantly. This shows that the heat treatment itself did not induce any selective mortality with respect to sex of the larvae. In rescued ghost adults, sex allocation is again extremely female biased, albeit less so than for larvae. This suggests that the mechanisms governing sex allocation in larvae and adults are different. The occasional male larva is a developmental relic, remnant of the transition from cyclical parthenogenesis to paedogenesis. Under physiological conditions, the rare male larvae do not develop into pupae or adults but die as larvae. The sex ratio of larvae has no functional meaning and is an artefact, whereas the sex ratio of the ghost adults might reflect the sex ratio that was operational before the species became obligately paedogenetic. The observed sex ratio variation in ghost adults across different locations, as opposed to larvae, might be indicative of the genetic isolation of the paedogenetic populations and of the consequential stochastic differences in the degree of vestigialization of the ghost adults.

There are a number of explanations for biased sex ratios, especially for species that live in rotting wood or other enclosed environments as is the case for *Micromalthus* where interactions among kin may be important^17^.

First, local mate competition (LMC) selects for female-biased sex ratios when related males (*i.e.* brothers or half-brothers) compete to fertilise their sisters^27^. By rescuing adults and observing their behaviour, it seems unlikely that LMC has been a source of sex ratio selection in *Micromalthus*. Most importantly, females have been observed to avoid mating with kin, exhibiting rejection behaviours and being less likely to mount males from the same log, which would reduce LMC. Males expose their aedeagus as soon as they emerge from the pupal stage, and despite being surrounded by females from the same patch, to which they are likely related, they are ignored; they then carry out a short flight towards a new patch of unrelated females (thus supporting the hypothesis of females choice). In addition, male premating dispersal limits any effect of LMC to either partial LMC or ameliorating it entirely if all matings are away from the natal log such that kin would rarely be involved in competition.

Second, local resource competition (LRC) can also select for biased sex ratios, although typically LRC involves competition amongst females for resources. LRC should select for male-biased sex ratios, which is clearly not the pattern observed in *Micromalthus* adults.

Third, local resource enhancement (LRE) can select for biased sex ratios if the offspring of one sex increases the fitness of parents. By feeding on their mother, there will probably be competition among female larvae, arguing against a LRC interpretation. As such, the biology of *Micromalthus*, including our novel observations of adult mating behaviour, does not offer strong support for LMC, LRC or LRE shaping the patterns of sex allocation observed.

Most non-cyclically haplodiploid species have deviating sex ratios as adults. Haplodiploid species with a social life history are almost all female biased. Often females control sex allocation directly. Most non-social haplodiploid species are parasitoids and have sex ratios ranging from highly male-biased^28^ to highly female-biased^29^. In sexually reproducing diplodiploid species, sex ratio biases of intrinsic genetic origin (e.g., not due to manipulation by endosymbionts) are extremely rare^30^. However, haplodiploid cyclically parthenogenetic species have often been overlooked. West^17^ suggested that haplodiploid cyclical parthenogens in particular might prove useful for testing Düsing and Fisher’s theory of sex allocation. Monogonont rotifers, cecidomyid midges, cynipid wasps and *Micromalthus* all fall into this special group. Despite their taxonomic disparity, these groups exhibit similar life cycles. Exemplified by rotifers, laboratory and field population studies revealed an even sex ratio for haplodiploid cyclical parthenogens over time^31^. Biases in haplodiploid cyclical parthenogens might be more informative than generally assumed. The three canonical explanations for sex ratio biases detailed above do not seem to be applicable to haplodiploid cyclical parthenogens and *Micromalthus*. So what might have caused the sex ratio deviation in *Micromalthus* adults?

### Mating behaviour of ghost adults is indicative of ancient sex-role reversal

The mating behaviour of our ghost adults reveals clear patterns of sex-role reversal, even when adults originate from different populations. Females initiated interactions, competed for mates, and even grasped the male genitalia with their own genitalia, leading in one case to male injury. Female-female competition was also more common when preferred mates (*i.e*. males from different patches) were available. Females are therefore both competitive over mates and also selective, avoiding (presumed) kin. This is another clear sign of choosy haplodiploid females, likely avoiding inbreeding^32^. However, female dance, which we considered a sort of ‘kin’ signalling behaviour, was more likely to occur with males from the same patch, suggesting that it is not courtship but rather another form of rejection behaviour by females. Our results therefore not only shed light on the sex ratio bias of *Micromalthus*, they also confirm that sex roles are not necessarily fixed, and support mating systems theory that predicts both males and females may combine choosiness and competitiveness over mates to some extent^33^,^34^.

### Vestigialization of adults and the loss of sexual reproduction

Are the adults indeed ghosts and the observed behaviours ancient? Leaky asexuality or rare sex in a predominant asexual species could upset any interpretation of the results. Most cases of cryptic or occasional sexual reproduction involve thelytokous species that have lost sexual reproduction. In cyclically parthenogenetic species becoming paedogenetic, the loss is two-fold: sex as well as the adult stage. In cyclical parthenogenesis the two are linked, however, in neotenic species, the two phenomena are independent of each other. For example, in ambystomid salamanders such as the Mexican axolotl, only the imago stage has been lost for both sexes. Sexual reproduction continues at a juvenile stage, in this case in an aquatic environment instead of in a terrestrial one. In most twisted wing insects (Strepsiptera) or certain scale insects and mealybugs (Hemiptera), the typical adult stage has been lost only for females but not for males, and sexual reproduction has become asymmetrical in the sense that adult males mate with females as larvae or pupae. In *Micromalthus*, both sexual reproduction and (virtually) the adult stages have been lost for both sexes.

The mechanism by which sexual reproduction is lost is of great importance^35^. Diplodiploid cyclically parthenogenetic aphids might lose sex through alterations of periodicity genes or genes that regulate hormonal expression^36^. This can happen so easily that a third of aphid species might be combinations of cyclically parthenogenetic and obligate asexual populations^37^. Diplodiploid cyclically parthenogenetic waterfleas (Cladocera) lose sex either through hybridogenesis or through a meiosis suppressor gene. Especially in the case of a meiosis suppressor gene, sex could return at any time and re-disappear thereafter. A sex-dependent meiosis suppressor gene cannot maintain itself in haplodiploid organisms^38^,^39^, which rules out any of these mechanisms for *Micromalthus*.

The dissection of ghost adults provides evidence that *Micromalthus* can no longer return to sexual reproduction. The adults are physiologically incapable of reproducing^10^,^11^,^12^,^18^. This has been experimentally confirmed and endorsed many times^9^,^40^,^41^. In the original species description based on adults, LeConte referred to the whole species as feeble and ill-developed^42^. This strongly suggests that *Micromalthus* lost the sexual part of its life cycle a long time ago, allowing for the vast degeneration in the rare ghost adults. The retention of functionless males in otherwise asexual species is well documented, but the retention of sterile females has so far been overlooked^40^. Hebert^40^ would consider the production of sterile females as “without precedent”. Indeed, the adult females we produced and rarely observed in the field (DKY) are ghost adults. It is well accepted that adults have disappeared completely in many now obligate parthenogenetic species^43^. It also indicates that rare episodes of sexual reproduction are unlikely to contribute to the survival of the observed behavioural pattern or *Micromalthus* as a species.

When sexually reproducing insect species become infected with parthenogenesis-inducing *Wolbachia*, vestigialization of males has been observed^44^,^45^,^46^. A characteristic of decay of sexual functionality in *Wolbachia*-induced thelytokous females of parasitoid wasps is the degeneration of the spermathecae^47^,^48^,^49^ as we found in the adults of *Micromalthus*. The extent of vestigialization depends on the age of the association. When *Wolbachia*-infected thelytokous females of various species are treated with antibiotics or heat to kill *Wolbachia*, a continuum ranging from fully functional males to no male production is observed^50^. It seems that behavioural functions like mate recognition are lost before physiological functions like male fertility disappear, or male production ceases altogether. In *Micromalthus*, behavioural functions including mate recognition are still strongly expressed whereas physiological functions have severely degenerated or have been lost altogether. This suggests that either the behaviour of ghost adults is more recent than we assume or that the cost of retaining the behaviour is different in every species; we favour the latter explanation. Nearly neutral behavioural traits might undergo very little decay even after extended periods of relaxed or absent selection^43^,^46^.

### Unifying features of *Micromalthus* and haplodiploid cyclical parthenogens

Behaviour of lost adults can be reconstructed only in a very small time window. According to fossil records (amber preserved triungulins), cyclical parthenogenesis is at least 112 million years old in *Micromalthus*^51^,^52^. The age of paedogenesis in cecidomyid midges has been estimated to be at least 30 million years based on Mexican amber and as old as 145 million years based on Canadian amber^53^. For *Micromalthus*, when functional adults were lost cannot be said yet. In the cyclically parthenogenetic rotifer, *Branchionus calcyciforus*, loss of sex and adults can easily be induced experimentally in the laboratory after 20–30 generations^39^. For insects, it seems to be a much slower process. Several species of the haplodiploid, cyclically parthenogenetic cecidomyid midges living in decaying tree bark and mushrooms, and cynipid wasps living in galls have lost sexual reproduction and the adult phenotype long ago. Two species, the midge *Heteropeza pygmaea*^54^,^55^,^56^ and the gall wasp *Andricus quadrilineatus* Hartig/*A. kiefferi* Pigeot^57^,^58^ seem to be at a similar junction in the evolution of their life cycles. Both species exhibit some striking similarities to the life cycle of *Micromalthus*. The two species produce adults only occasionally, mainly after artificial induction. They exhibit hemocoelous development of the larvae inside the hemocoel of the mother larvae, while *Micromalthus* exhibits matriphagy. These two species have an extreme female bias and adults that are increasingly sterile, while in *Micromalthus* sexual reproduction has disappeared altogether. Both species also show large regional variation in the number and relative sterility of adults produced. The midge, wasp and beetle (*Micromalthus*) have already undergone a functional transition from a cyclically parthenogenetic metamorphosis to a paedogenetic life cycle. Any adults are induced in paedogenetic larvae and develop from paedogenetic larvae. We propose that the sex ratio of these ghost adults is the result of the paedogenetic development. We also propose that all haplodiploid cyclically parthenogenetic species that transition to a paedogenetic life style will exhibit a female bias.

### Probable role of endosymbionts in *Micromalthus*

Perhaps the sex-role reversal we observed was the result of reproductive parasites^59^,^60^,^61^. Sex ratio distortion caused by bacteria or parasites, often referred to as endosymbionts, is widespread in arthropods; examples of microbes that manipulate the reproduction of their haplodiploid beetle hosts are well known^62^,^63^.

*Rickettsia bellii* endosymbionts have been detected in all analysed larvae suggesting they might serve an obligate beneficial role in the larvae which might include restoring diploidy in oocytes during parthenogenetic reproduction as in psocids^64^. Assuming for the purpose of argument that the *Rickettsia* do restore diploidy in *Micromalthus*, then the heat required to induce males must be high enough to disable or kill the *Rickettsia* so that haploid male larvae can develop. Indeed, *Rickettsia* cannot be detected in adults. However, this would also postulate that the current form of paedogenesis of *Micromalthus* must be different from the ancestral, holocyclical form of paedogenesis, which we assume was not *Rickettsia* infected. Alternatively, these *Rickettsia* may provide a benefit other than restoring diploidy. Interestingly, *R. bellii* has been associated with male killing in a buprestid beetle^63^.

*Wolbachia pipientis* endosymbionts were detected in only 5% of the larvae, which argues against a beneficial association or a still functional association. The absence of the bacteria from the adults is likely a direct consequence of the heat treatment and as such an epiphenomenon. The *Wolbachia* bacteria seem now to be on a trajectory of being lost stochastically. It is possible that the *Wolbachia* infection is a vestige of a sex ratio distortion event, although it does not rule out that these *Wolbachia* might have been beneficial to the adult stage at one time and are now superfluous. In *Acraea* butterflies, male-killing *Wolbachia* can exert such selection pressure upon a species that the endosymbionts can cause sex-role reversal^65^. Selection pressure can lead in some species to rapid suppression of the male-killing effect^66^, while other species are unable to respond to the male-killing effect^67^. The *Wolbachia*-induced extreme shortage of males in the blue moon butterfly, *Hypolimnas bolina*, precipitates female promiscuity^68^. We see in *Micromalthus* ghost adults the strong maintenance of mate choice that discriminates on the basis of kin selection despite an extreme shortage of males. This suggests that in a given species female response to male scarcity is not predictable. Endosymbionts might explain a female bias and behaviour in *Micromalthus* while it was a sexually reproducing species; endosymbionts cannot explain the observed sex ratio in ghost adults.

Unlike the biases controlled by sex allocation mechanisms and selection, the female bias of ghost adults is probable a temporary by-product of asexual reproduction at a juvenile stage.

By reconstructing adults of an ancient haplodiploid cyclical parthenogen, we have shown that they have strongly female-biased sex ratios in ghost adults. Our behavioural observations of these ghost adults show remarkable sex-role reversal behaviour. We hypothesize that the observed female-biased sex ratio is a physiological consequence of paedogenetic difficulties of producing male offspring, likely characteristic to all haplodiploid cyclical parthenogenetic species transitioning to a paedogenetic life cycle. Unrelated to the female bias of currently induced adults, the recovered behaviours are the consequence of an ancient female-biased sex ratio. This ancient female bias predates the loss of adults and might have been induced by a male-killing sex ratio distorter.

## Methods

### Colonies of *Micromalthus debilis*

Oak logs containing colonies of *M. debilis* were collected by DKY in 2002, 2004, 2005 and 2006 from two sites (43.19361°N, −90.23917°W; 43.19360°N, −90.23844°W) in Richland County, 46 miles west of Madison, Wisconsin (USA). Logs from these two sites provided colonies from five different locations. Colonies are separate patches on logs. Logs were reared for five years at Bangor University, kept under constant environmental conditions inside styrofoam boxes (18 ± 2 °C and 90% RH). The location descriptors are: Location 1, 2002; Location 2, 2004; Location 3, 2006a; Location 4, 2005; and Location 5, 2006b.

### Manipulation of specimens

The *Micromalthus*-containing wood is tunnelled by larvae parallel to the trunk and up to 2.5 cm deep into the bark. It allows fragmentation using microdissection forceps. Although this method is disruptive it is the only way to reach, observe and collect the small, fragile specimens (legged triungulins reach up to 1 mm, while mature cerambycoids attain a length of up to 3.3 mm)^52^. Due to their small size, all procedures were performed under magnification lenses and stereomicroscopes. Whole colonies were isolated in insect containers.

### Heat treatment and the induction of ghost adults

In February 2005, five, 2004 colonies were exposed to a high temperature treatment (HT^12^: with the photoperiod fixed at 16 h light. The treatment consisted of progressively raising the temperature inside the cages containing *Micromalthus* over the course of a month, to a daily maximum of 55 °C at mid-afternoon and a night minimum of 20 °C for 3 weeks (55% RH). The colonies were daily monitored every two hours for the presence of adults. Once adults started emerging (March 2005), they were monitored every hour and counted. Sexing was performed *in situ* and controlled later by examining preserved specimens under the stereomicroscope. The treatment was repeated in March 2007 with two colonies from each of the 2004, 2005 and 2006a and b collections (*i.e.* N = 8 colonies). Adults emerged from only five of these eight colonies; the other three colonies never produced adults as a result of HT.

### Sex ratio of larvae

To estimate the sex ratio, samples of wood were disaggregated and the larvae counted and sexed in two sets of 24 samples of approximately 2 cm^3^ wood. One of the two sets was exposed to a heat treatment (named After-HT), while the other set was called Before-HT. The controls consisted of populations kept at lab environmental conditions, not exposed to HT (named Controls). Controls allowed testing for natural variation, *i.e.* whether larvae died of natural causes, or other environmental modifications, other than heat and drought. Larval counting was destructive, because once the larvae are exposed to the elements, they can no longer be reintroduced into the tunnels and they die. The moment adults started emerging from the HT populations the wood pieces were opened and the larvae were counted.

### Behavioural assays

Three hundred and sixty seven adults (350 females and 17 males) were used in experiments of sexual behaviour. They were grouped in 17 sets of mixed sex, with experimental group named Same Patch (as the patch of the male) and Unrelated (different to the patch of the male) and six sets of single sex, female-only groups (experiments) named Controls. The grouping in sets was only governed by the patch and did not alter the sex ratio. All sets contained 6–21 individuals each (21 individuals was the maximum number that the observer could handle). Each session took place 30 min post-emergence of the adults, once adults were located inside the experimental area. Observations of behaviors lasted a maximum of 55 min because the beetles disappeared inside the wood chips, becoming inactive for the rest of their life span; males did not move anymore, some females flew away in the direction of the light. Several populations did not produce males, therefore the number of experiments on sexual behaviour were limited to the availability of non-impaired males (N = 17).

The behavioural experiments were conducted with pieces of wood containing *Micromalthus* from three of the five locations. Observations took place under two dissecting microscopes, using up to 40x (Leica ZOOM 2000) and 100x (Leitz, Wetzlar) magnification. Once adults commenced emerging, they did so daily, between 11:30 (earliest) and 17:00 (latest). Behaviours were studied on a specially designed arena consisting of an open glass-disc containing a 2 cm^3^-size fresh wood piece in the centre.

In each experiment, the male was carefully transferred on the tip of a smooth brush to the wood surface of another female-producing colony (patch of a log). Behaviours were scored by presence/absence of behaviour per female. Females were easily identified not only by size: some kept the wings extended in particular positions, others could not close the elytra, and most had different loads of nymphal phoretic Astigmata mites (these mites were seen solely on females).

### Behaviour descriptors

**A** – Male discrimination: Females ignored the presence of a male emerging from the same patch of wood. Females ran away from the male; on occasions they faced the males head on and moved their mandibles against the male.

**B** – Female-Female mounting: The females attempted to mount each other at a rate of one per minute. A mounting lasted 10 to 20 seconds (N = 4) (longer when a larger female was on top).

**C** – Female dance (Movie S1): Immediately after emergence females produced an up-down and sideways shaking of their abdomens while beating their wings.

**D** – Female-Male mounting: Females mounted the male.

**E**  – Female combat: Females fought with each other, trying to dislodge the female on top of the male, even piling on the male.

**F**  – Females injuring males: Rare behaviour where two females were observed to grasp the male genitalia leading to male injury in one case.

Images and movies were obtained by attaching with a tube adaptor (Brunel Microscopes) a digital camera (SONY Cyber-shot MP, 3x optical zoom) to one of the oculars.

### Life span, anatomy and physiology of ghost adults

All emerging adults used in the behavioural experiment were followed until death. The life span of 23 females and 12 males was measured; males were the limiting factor in these experiments.

The external morphology of all 1,059 adults was examined. The abdomen was screened with inverted light to detect signs of gross vestigialization and 10 of these were subsequently dissected.

In addition, the abdomen of 50 females involved in the behavioural experiments (plus 10 clearly physically impaired individuals) were dissected and the internal organs, particularly the ovaries, examined.

### Analysis for endosymbionts

*Micromalthus* larvae were screened for endosymbiosis: 10 individuals from location 2 were investigated by transmission electron microscopy, 60 by molecular analysis (PCR, sequencing), and 22 by fluorescent *in situ* hybridization (FISH) with species-specific probes (for confirmation of localization of relevant species). The 60 and 22 specimens were randomly picked from locations 2, 3, 4 and 5.

For FISH, whole mature cerambycoid larvae were fixed; fixation, hybridization, mounting and confocal microscopy analyses followed methods described in Perotti *et al.*^64^ for whole-specimen FISH on insects.

The 60 above described larvae and 60 non-impaired adult beetles (randomly picked from all adult producing colonies) were screened by PCR amplification using rDNA16S primers for sex ratio distorting bacteria and *wsp*A primers for *Wolbachia* following previously described protocols^30^,^64^.

### Statistical analyses

A binomial test checked for deviation from expected adult sex ratio (*i.e.* 0.5).

For paired sex ratios before and after HT, frequencies were transformed into proportions (male/(female + male) and compared with a non-parametric Wilcoxon Signed Rank test; to check for normality we used the Kolmogorov-Smirnov test.

To test for the effect of locality on the sex ratios Before and After HT, a Generalized Linear Model was fitted to a Poisson distribution with link log criteria, analysis type Wald and full likelihood ratios. Localities were analysed as main factors. The analyses were two-tailed; we used SPSS v. 22.

For adults, a Likelihood Ratio (LR) was calculated to measure the effect of location on sex ratio between different populations, and the effect of relatedness (Unrelated and Same Patch) between different behaviours. Generalised Linear Mixed Models were used for each of the six behaviours using a Poisson regression with link log to explore random effects of location and behaviour. A Zero-Inflated Poisson regression was initially run for overdispersed zero-inflated data, however, the Vuong test indicated no significant difference with the standard Poisson regression (STATA v. 13.1).

## Additional Information

**How to cite this article**: Perotti, M. A. *et al.* The ghost sex-life of the paedogenetic beetle *Micromalthus debilis*. *Sci. Rep.* **6**, 27364; doi: 10.1038/srep27364 (2016).

## Supplementary Material

Supplementary Video 1

Supplementary Information

## Figures and Tables

**Table 1:** Sex ratio of paedogens.

	HT Treatment and Controls					
	Before			After		
	FF	MM	SEX RATIO	FF	MM	SEX RATIO
Grand Total	5,007 (213)	24 (1)	0.0046 (±0.005)	2,521 (64)	10 (0)	0.002 (±0.004)
Treatments	2,502 (226)	11(1)	0.0044 (±0.005)	8 (0)	0	0
Controls	2,505 (210)	13 (1)	0.0046 (±0.006)	2,513 (214)	10 (1)	0.004 (±0.006)

Total number of females FF, total number of males MM (N_samples_ = 24 for Total and 12 for Treats and Controls; Median indicated between brackets), and averages of sex ratio [MM/(FF+MM)] obtained from each set of sub-samples (N_*samples*_ = 24 and 12; SD indicated between brackets).

**Table 2:** Adults that emerged from the wood of four locations after the heat and drought treatment (HT), except for the control.

Location/Origin	FF	MM	SEX RATIO
2 [n = 46]	483 (7)	4 (0)	0.005 (±0.02)
3 [n = 14]	224 (11)	37 (0)	0.135 (±0.28)
4 [n = 2]	47 (24)	0	0
5 [n = 13]	246 (12)	18 (0)	0.049 (±0.11)
Grand Total	1,000 (9)	59 (0)	0.037 (±0.13)

There were four Locations from which adults were counted [Number of emergences are indicated in square brackets]. Males and females were counted (Median indicated between brackets). Averages of sex ratio [MM/(FF+MM)] were obtained for each location or origin of the wood (SD indicated between brackets).
