# Supplementary material for: The ghost sex-life of the paedogenetic beetle Micromalthus debilis
Source: Sci Rep. 2016 Jun 7;6:27364. doi: 10.1038/srep27364 (PMC4895236; doi:10.1038/srep27364)
Supplement: Supplementary Information [file srep27364-s2.pdf]

SUPPLEMENTARY Materials

The ghost sex-life of the paedogenetic beetle *Micromalthus debilis*

M. Alejandra Perotti, Daniel K. Young, & Henk R. Braig

Supplementary figures 2
Movie snapshot and legend 4
Supplementary tables 5

SUPPLEMENTARY FIGURES

SUPPLEMENTARY FIGURE S1

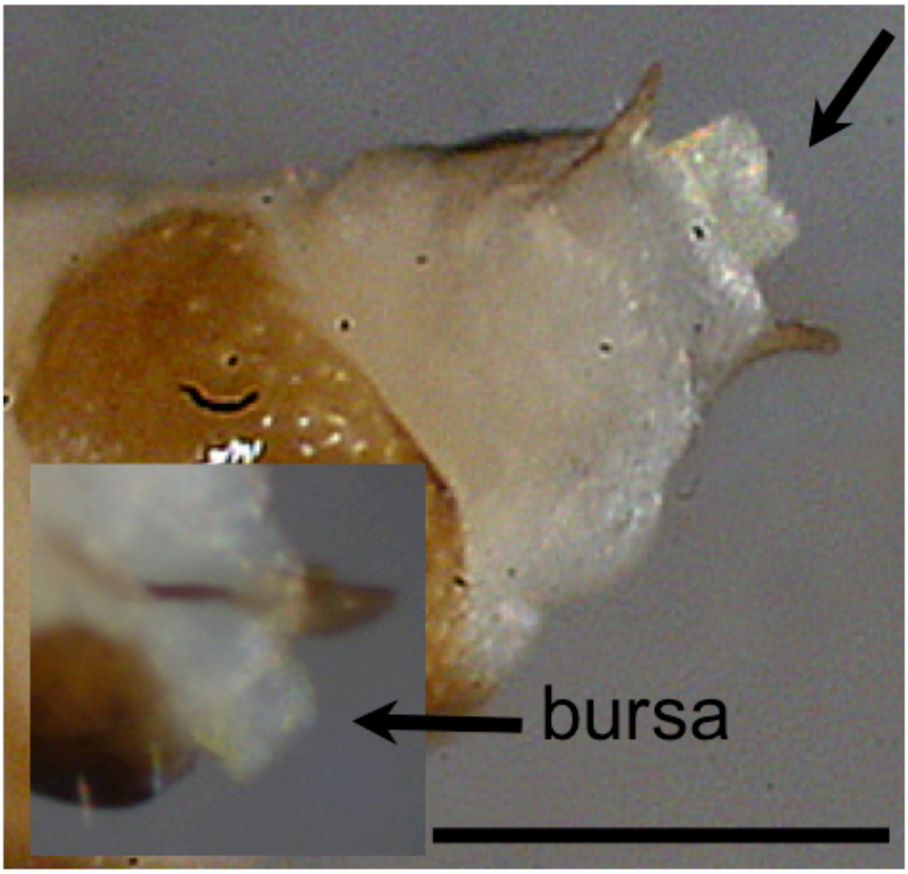

Everted *bursa copulatrix*, arrows showing the position of the *bursa* in the adult female (insert shows the bursa in lateral position) (Bar 100 μm).

SUPPLEMENTARY FIGURE S2

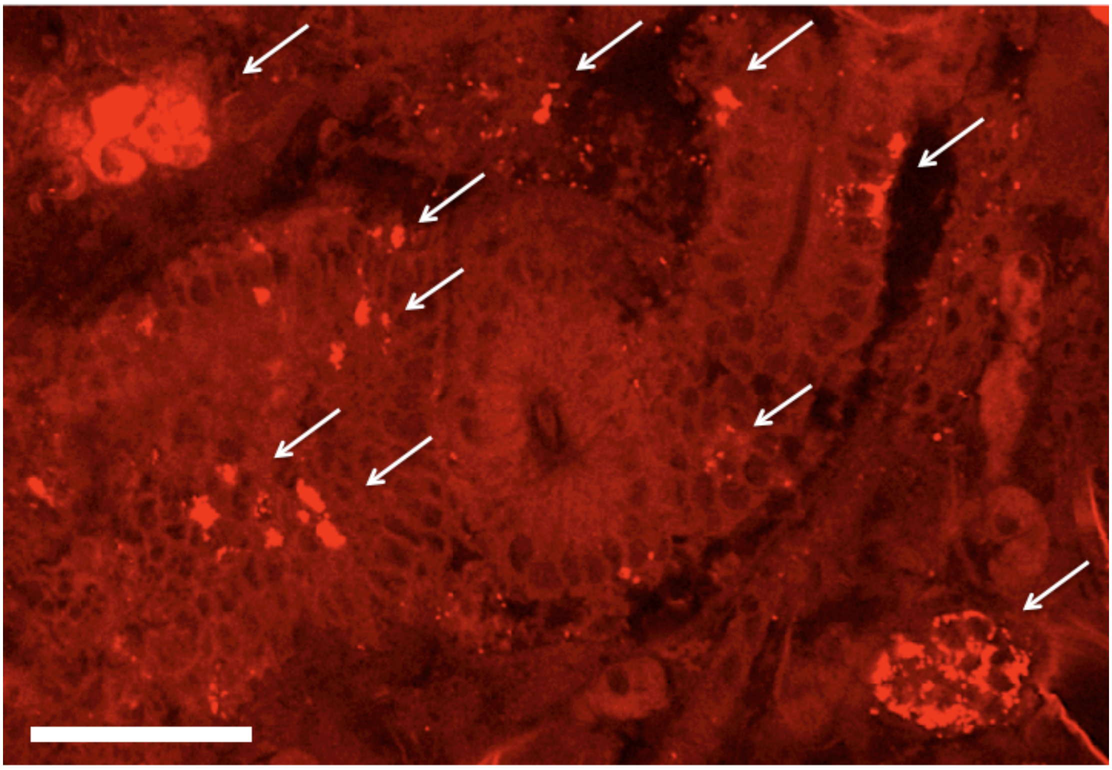

Rickettsia-specific FISH probe labelled with Cy5 on *Micromalthus* larvae showing oesophagus and midgut in the centre of the picture. Arrows point to *Rickettsia*-filled mycetocytes in the epithelium of the midgut and in the upper left corner and lower right corner a paired mycetomic organ (Bar 50 μm).

SUPPLEMENTARY MOVIE (SNAPSHOT)

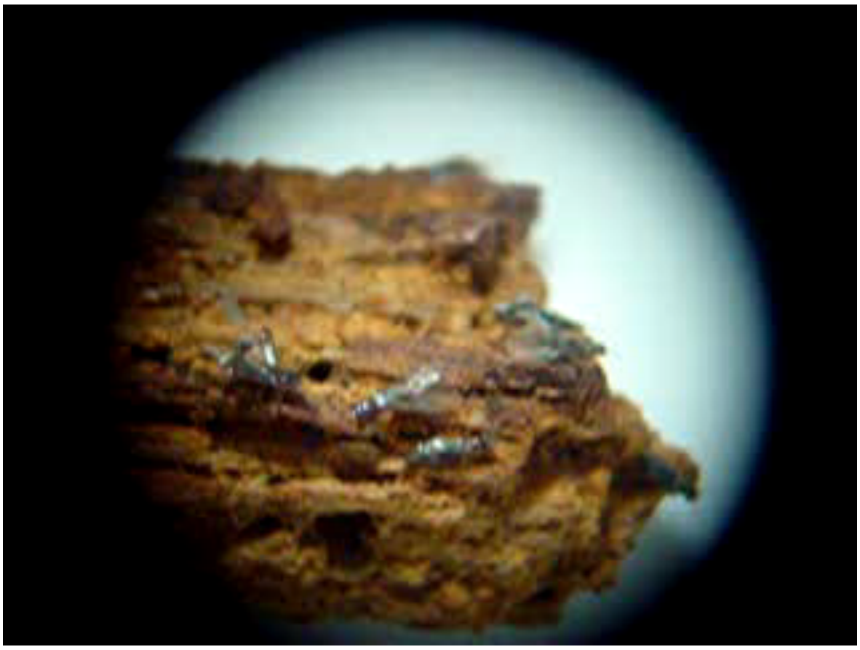

Movie legend

Movie S1: Female dance: Immediately after emergence from their pupae, females produce an up-down, sideways shaking of their abdomens while beating their wings.

SUPPLEMENTARY TABLES

Table S1: Primary sex ratios and effect of Heat (and drought) Treatment (HT) on *Micromalthus* larvae. Fourty-eight mature populations of larvae were used, of logs of different origins (defined Locations are found in Methods). Populations were used in 2 Sets of 2 x 12 populations each, Set 1: HT applied and Set 2: Controls or never exposed to HT. Larvae were counted before and after the HT, with Treat1 indicating Before and Treat2 indicating After exposure (or not, like for Set2). Larvae were extracted, sexed and counted in each of the two sets.

Sets	Locations	Treat 1 Females	Treat 1 Males	Treat 2 Females	Treat 2 Males
1-HT applied	1	235	0	0	0
1-HT applied	1	257	1	0	0
1-HT applied	2	229	1	0	0
1-HT applied	2	258	1	0	0
1-HT applied	3	178	3	1	0
1-HT applied	3	111	0	0	0
1-HT applied	3	199	0	7	0
1-HT applied	3	127	1	0	0
1-HT applied	4	243	1	0	0
1-HT applied	4	222	2	0	0
1-HT applied	4	251	1	0	0
1-HT applied	3	192	0	0	0
2-NO HT	2	200	0	235	1
2-NO HT	2	250	0	199	0
2-NO HT	1	233	2	226	0
2-NO HT	1	271	1	249	2
2-NO HT	3	156	1	121	0
2-NO HT	3	209	0	173	0
2-NO HT	3	177	0	184	1
2-NO HT	3	134	0	129	1
2-NO HT	4	216	3	201	4
2-NO HT	4	210	1	266	0
2-NO HT	4	263	5	298	1
2-NO HT	3	186	0	232	0

Table S2: Pairs of datasets to compare *Micromalthus* larva and adult sex proportions by locations (the only populations of larvae and adults that could be paired, according to Location).

Sex	Replicate	Location	Larvae Control	Larvae After-HT	ADULTS
Females	1	2	235	0	72
Females	2	2	199	0	200
Male	1	2	1	0	1
Male	2	2	0	0	0
Females	1	3	121	0	132
Females	2	3	173	0	59
Male	1	3	0	0	6
Male	2	3	0	0	31

Table S3: Results of Behavioural observations of *Micromalthus* adults. Three Groups of females (called Experiments) were used. The origin of each Group set was also controlled (Locality). Behaviours: A: Male discrimination; B: Female-female mounting; C: Female dance; D: Female-male mounting; E: Female-female combat; and F: Females injuring males.

Experiments	Number of Females	Behaviours A	B	C	D	E	F	Locality
Related	17	17	9	6	0	0	0	2
Related	18	18	8	8	0	0	0	2
Related	18	18	9	10	0	0	0	2
Related	15	14	11	8	0	0	0	3
Related	15	7	10	13	4	3	0	3
Related	20	20	14	19	0	0	0	3
Related	20	20	6	14	0	0	0	5
Related	20	18	10	17	0	0	0	3
Related	12	12	12	11	0	0	0	3
Not related	9	0	9	0	8	6	2	2
Not related	14	0	7	0	7	5	0	2
Not related	20	4	16	10	15	7	0	3
Not related	20	0	4	3	14	11	0	3
Not related	20	0	5	6	17	13	0	3
Not related	17	0	2	5	12	9	0	3
Not related	17	2	11	7	17	15	0	5
Not related	20	0	17	8	17	17	0	3
Controls	6	0	5	6	0	0	0	2
Controls	9	0	6	8	0	0	0	2
Controls	11	0	5	11	0	0	0	2
Controls	10	0	4	10	0	0	0	3
Controls	14	0	5	13	0	0	0	3
Controls	8	0	3	8	0	0	0	5
